# Design and ICH validation of a stability-indicating green RP-HPLC method for 5,6,7-trihydroxyflavone with multi-metric greenness evaluation and application to nanopharmaceutical systems

**DOI:** 10.1039/d6ra01533f

**Published:** 2026-04-13

**Authors:** Sweta Acharya, Ankit Jain

**Affiliations:** a Industrial Research Laboratories, Department of Pharmacy, Birla Institute of Technology and Science Pilani Campus Pilani Rajasthan 333031 India ankit.j@pilani.bits-pilani.ac.in

## Abstract

A novel stability-indicating reverse-phase high-performance liquid chromatography (RP-HPLC) method was developed and validated for the quantification of baicalein (5,6,7-trihydroxyflavone) in bulk drug and nanoliposomal formulations. Samples were pretreated *via* dilution with mobile phase, membrane filtration (0.22 µm), protection from light, and refrigerated storage prior to analysis. Method validation was performed in accordance with ICH Q2(R2) guidelines. Chromatographic separation was achieved on a BDS C18 column using an isocratic mobile phase comprising water (pH 3.0, adjusted with orthophosphoric acid) and acetonitrile (50 : 50, v/v) at a flow rate of 1.0 mL min^−1^ with DAD detector. The method exhibited excellent linearity (0.0078–1 µg mL^−1^; *R*^2^ = 0.9998), accuracy (98.25–101.58%), and precision (%RSD < 2%). The limits of detection (LOD) and quantification (LOQ) were 0.0069 µg mL^−1^ and 0.0208 µg mL^−1^, respectively. Forced degradation under acidic, alkaline, oxidative, and thermal stress conditions confirmed the stability-indicating capability, with pronounced degradation under acidic conditions and high thermal stability. The method was successfully applied to solubility profiling and quantification in nanoliposomal systems, including entrapment efficiency and drug loading. Greenness assessment using Blue Applicability Grade Index (BAGI), Modified Green Analytical Procedure Index (MoGAPI), Carbon Footprint Reduction Index (CaFRI), Click Analytical Chemistry Index (CACI), Analytical GREEnness (AGREE), and Violet Innovation Grade Index (VIGI) yielded scores of 75, 85, 77, 80, 0.64, and 55, respectively. The method demonstrates key advantages, including buffer-free operation, reduced chemical hazard, and operational simplicity; however, moderate organic solvent usage imposes minor sustainability limitations. Overall, the method is robust, sensitive, and suitable for routine pharmaceutical and nanoformulation analysis.

## Abstract 2

Buffer-free, stability-indicating RP-HPLC enables rapid analysis of baicalein. Integrated greenness metrics and nanoformulation applicability establish a simple, sustainable, and versatile analytical platform.

## Graphical abstract

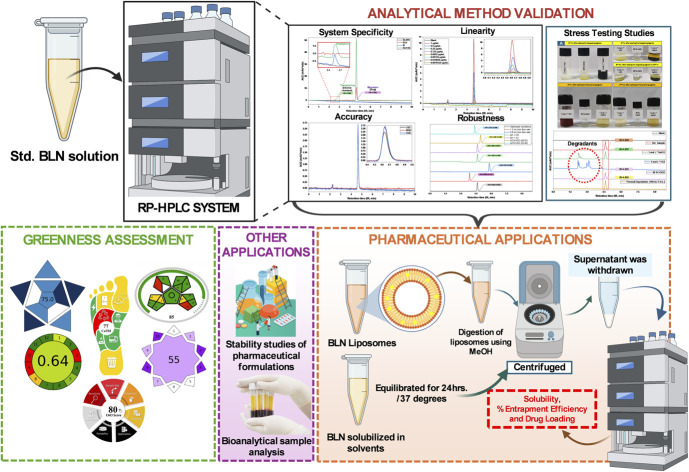

## 1. Introduction

High-performance liquid chromatography (HPLC) is widely used in pharmaceutical analysis for its efficiency in analyzing multiple compounds, including associated impurities and degradants. The use of this technique has increased in recent years across laboratory and industrial settings due to its precise, accurate determination of analytes of interest. This technique proves itself as an efficient method for determining impurities, especially in Active Pharmaceutical Ingredients (APIs), which is a significant task, as even trace amounts of these can lead to side effects, toxicity, or reduced efficacy of the drug over time.^1,2^

In analytical method development, appropriate sample pretreatment is a critical step to ensure accuracy, reproducibility, and compatibility with chromatographic systems. Common strategies include dilution with suitable solvents, membrane filtration (0.22 µm), and protection from environmental factors such as light and temperature to prevent analyte degradation and matrix interference. These steps are particularly important when dealing with complex pharmaceutical matrices and nanocarrier-based systems.

For any analytical method to be robust, it must be validated to comply with international regulatory standards and demonstrate its suitability for use. According to the International Conference on Harmonization (ICH) Q2(R2) guideline, a validated quantitative analytical procedure is capable not only of quantifying the drug in pharmaceutical formulations but also of detecting changes in product quality during storage, which can be confirmed if a stability-indicating test is able to separate the drug from its degradation products.^3^

In this context, forced degradation studies are essential to establish the stability-indicating capability of an analytical method. Such studies involve subjecting the analyte to stress conditions, including acidic, alkaline, oxidative, and thermal environments, to evaluate degradation behavior and ensure effective separation of degradation products from the parent compound.

**Fig. 1:**
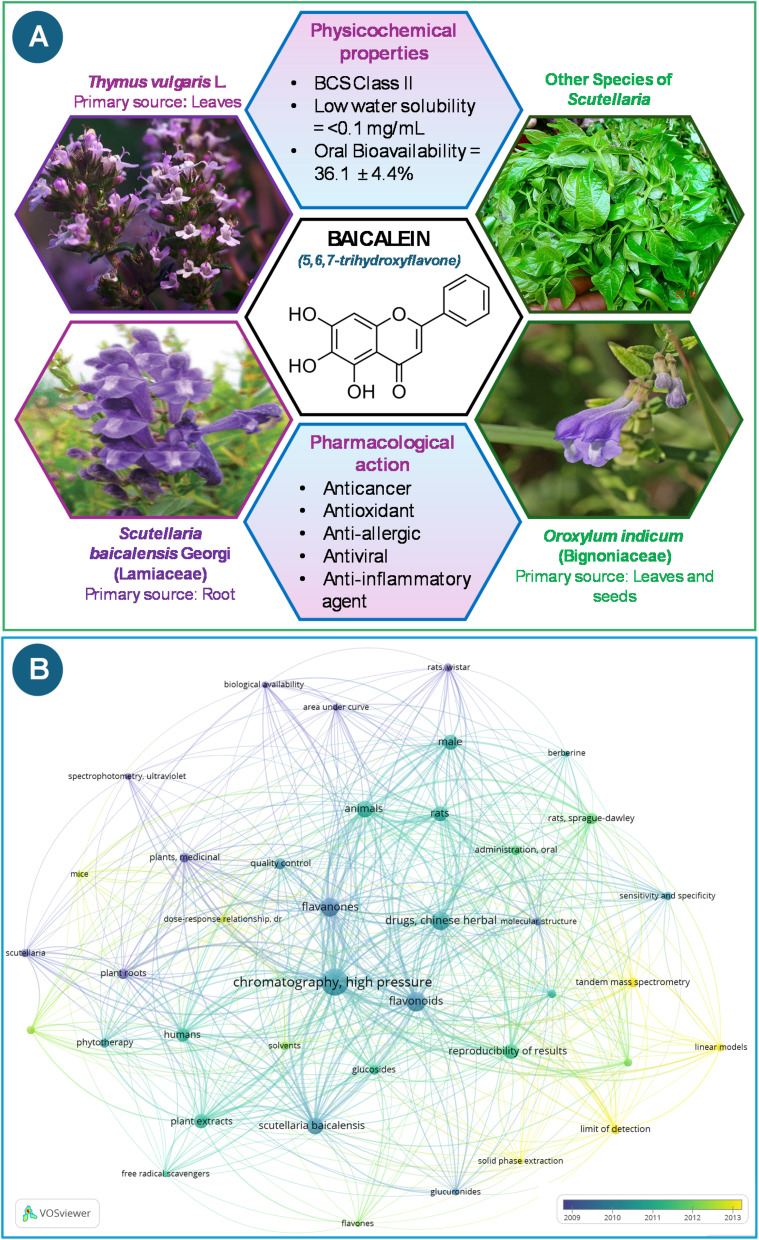
(A) Sources, challenges, pharmacological action, and chemical structure of baicalein, and (B) bibliometric analysis representing research in the method development of baicalein and other plant extracts.

Baicalein (BLN) is a 5,6,7-trihydroxyflavone (Fig. 1(A)), which is obtained from the roots of *Scutellaria baicalensis* Georgi (Lamiaceae) and is widely used in Traditional Chinese Medicine. Recently, it has been classified as a Biopharmaceutics Classification System (BCS) Class II substance because of its low water solubility (<0.1 mg mL^−1^), which causes an oral bioavailability (BA) of 36.1% ± 4.4% and restricts its medicinal use.^4,5^ BLN has a wide range of pharmacological activities, including anticancer, antioxidant, anti-allergic, antiviral, and anti-inflammatory properties.

So far, *in vivo* studies have shown that BLN can efficiently reduce inflammation associated with cancer, neurodegenerative diseases, and other conditions. As the clinical translation of BLN accelerates, the development and validation of this drug have become increasingly important, underscoring the need for a precise and trustworthy analytical technique (Fig. 1(B)).^6^

Current HPLC methodologies for quantifying BLN use a phosphate-buffered mobile phase, multi-component gradient elution, or a high percentage of organic solvent to control phenolic compound ionization and improve peak symmetry.^6,7^ Although these approaches are effective in achieving the desired chromatographic resolution, they are associated with considerable environmental consequences. The use of a buffer-based mobile phase results in the generation of waste containing inorganic salts that need careful treatment and disposal, causes column fouling and reduces the column's lifespan, and increases the solvent consumption and analysis time during column re-equilibration. The use of a multi-component gradient elution approach also increases the environmental impact of the analysis due to excessive solvent consumption during column re-equilibration.

Recent advancements in pharmaceutical analysis emphasize the adoption of sustainable and eco-friendly analytical methodologies. Multi-metric greenness assessment tools such as the Blue Applicability Grade Index (BAGI), Modified Green Analytical Procedure Index (MoGAPI), Analytical GREEnness (AGREE), Carbon Footprint Reduction Index (CaFRI), Click Analytical Chemistry Index (CACI), and Violet Innovation Grade Index (VIGI) enable comprehensive evaluation of environmental impact, method efficiency, and innovation. These tools are increasingly integrated into method development to ensure compliance with Green Analytical Chemistry principles.

In addition, high-performance liquid chromatography coupled with diode-array detection (HPLC-DAD) provides enhanced analytical capabilities through spectral peak purity analysis, improved selectivity, and reliable quantification, making it particularly suitable for stability-indicating studies and complex pharmaceutical formulations. Recent eco-friendly HPLC-DAD methods have demonstrated significant potential in reducing environmental burden while maintaining analytical performance.

In contrast, the current approach uses a mobile phase consisting of Milli-Q water at pH 3, adjusted with orthophosphoric acid, and acetonitrile under isocratic conditions. This approach avoids the generation of inorganic salts and the use of auxiliary reagents. Moreover, the current approach avoids ionic-strength-based control in the analysis of baicalein and instead controls the compound's ionization by adjusting the pH of the mobile phase. As a result, the current approach has a lower environmental impact than the previously published methodologies and can be regarded as a ‘greener’ approach to the analysis of baicalein.

Furthermore, the increasing use of nanopharmaceutical systems, such as liposomes and lipid-based carriers, necessitates robust analytical techniques for accurate quantification, drug loading, entrapment efficiency, and stability assessment, thereby reinforcing the need for sensitive and reliable chromatographic methods.

Therefore, the aim of this work was to develop and validate a sensitive, stability-indicating, and environmentally sustainable RP-HPLC method for the determination of BLN in accordance with ICH guidelines. The proposed method integrates buffer-free chromatographic conditions, multi-metric greenness evaluation, and applicability to nanopharmaceutical systems. The method offers significant advantages, including reduced hazardous waste generation, operational simplicity, and high sensitivity; however, the use of organic solvents poses a minor limitation for overall sustainability.^8–10^

## 2. Experimental

### 2.1. Materials

Baicalein (BLN, purity ≥ 98%, analytical grade, CAS no. 491-67-8), a flavonoid with the IUPAC name 5,6,7-trihydroxy-2-phenylchromen-4-one, was obtained from Density Pharmachem Private Limited (India). The supplier provided a certificate of analysis confirming purity and identity. The compound was stored in a desiccated, light-protected environment at 2–8 °C to prevent degradation. Acetonitrile (ACN, HPLC grade) was obtained from Qualigens Pharma Private Limited (India). De-ionized water was prepared by reverse osmosis using a Millipak system (0.22 µm; Millipore, MA, USA). Reagents such as hydrochloric acid (HCl), sodium hydroxide (NaOH), orthophosphoric acid (OPA), and hydrogen peroxide (H_2_O_2_) were obtained from Sisco Research Laboratories (India). Theophylline was purchased from Tokyo Chemical Industry Co., Ltd. The LIPOID S PC-3 (phosphatidylcholine, hydrogenated, content ≥ 98.0%), CAS-no. 97281-48-6, was provided as a gift sample from Lipoid® GmbH, Ludwigshafen, Germany. Cholesterol was purchased from Sigma-Aldrich Corporation.

### 2.2. Instruments and software

The analytical method was developed on the Thermo Scientific™ UltiMate™ 3000 Standard Quaternary Analytical System manufactured by Thermo Fisher Scientific. Data acquisition was performed using Chromeleon 7.2 SR4 Software. The particle size analysis and zeta potential analysis were performed using the Malvern Zetasizer Nano ZS, manufactured by Malvern Panalytical. The data on particle size and zeta potential were obtained using the Zetasizer software. The purification of liposomes was performed by repeated washing and centrifuging the solution using an Eppendorf 5415R centrifuge (Eppendorf AG). The UV spectrum was obtained using the Shimadzu UV-2600 (or the updated UV-2600i) single-monochromator ultraviolet-visible spectrophotometer, manufactured by Shimadzu Corporation. The UV-spectral data were obtained using the UVProbe 2.61 software. Graphical representations and statistical tests were generated using OriginPro 2026 (learning edition). Diagrams were created using Biorender software under an academic license.

### 2.3. Preliminary investigation: ultraviolet (UV) spectroscopic determination of baicalein

The UV spectroscopic method was adapted from previously reported procedures for flavonoid analysis with minor modifications. For the preparation of the working solution aliquot of BLN, the primary stock solution in methanol at 1 mg mL^−1^ was diluted to 10 µg mL^−1^. The UV spectra were obtained by placing the prepared solutions into a quartz cuvette. The spectral information was acquired using a Shimadzu 1800 spectrophotometer over a wavelength range of 200–400 nm, with methanol as the blank.^11^

### 2.4. Instrumentation and chromatographic conditions

The Thermo Fisher Ultimate 3000 system, integrated with a quaternary pump, a degasser unit with a 100 µL autosampler loop, and a diode-array detector (DAD), was used to develop the method. Data acquisition was performed using Chromeleon 7.2 SR4 Software.

Optimization of chromatographic conditions was performed using a univariate (one-factor-at-a-time) approach, systematically varying mobile-phase composition, pH, flow rate, and detection wavelength to achieve optimal resolution, peak symmetry, and retention time. Method performance was evaluated based on retention time (tR), peak area, tailing factor, and theoretical plate count. Quantification of BLN was carried out using peak area as the analytical response. Matrix effects were minimized through appropriate sample pretreatment and dilution; no significant interference was observed at the BLN retention time, confirming method specificity. The optimized analysis parameters are given in Table 1. The optimization of chromatographic conditions focused on the mobile-phase ratio, isocratic profile, injection volume, analysis time, and column temperature.

**Table 1:** Optimized chromatographic conditions for quantifying BLN

Parameters	Description
Instrument	Thermo Fisher Ultimate 3000 HPLC system equipped with a quaternary pump, autosampler, column oven, and DAD
Column	BDS C18 Reversed-phase HPLC column, 5 µm, 4.6 mm × 250 mm
Mobile phase	Water (pH 3.0 ± 0.05, adjusted with OPA) : ACN, 50 : 50 (v/v)
Flow rate	1.0 mL min^−1^
Detection wavelength	200–400 nm (*λ*_max_of BLN = 275 nm)
Injection volume	20 µL
Column temperature	Ambient or 25 °C
Autosampler temperature	10 °C
Run time	10 min
Diluent	Mobile phase (50 : 50 v/v)
Elution	Low-pressure isocratic mode
Pressure	1195–1208 psi

### 2.5. Preparation of calibration standards

The previously prepared primary stock solution of BLN was used. The solution was protected from light and refrigerated (4 ± 0.5 °C) until further analysis, which was then diluted to make secondary stocks at 100, 10, and 1 µg mL^−1^ using the same solvent medium. Working solutions were prepared by diluting the 1 µg mL^−1^ solution with mobile phase to produce calibration standards at concentrations of 1, 0.5, 0.25, 0.125, 0.0625, 0.0312, 0.0156, and 0.0078 µg mL^−1^.

### 2.6. Validation of analytical method

The current method was validated for system suitability, specificity, linearity, precision, accuracy, and robustness in accordance with ICH Q2 (R2) (1 November 2023), as described below.

#### 2.6.1. Specificity

Specificity is the ability to unequivocally assess the analyte in the presence of components that may be expected. Typically, these might include impurities, degradants, matrix, *etc.* The method's specificity was validated by injecting four samples: the blank (placebo), theophylline (internal standard), baicalein (drug), and a mixture of both. The chromatogram obtained, should not show any interfering peaks at the BLN retention time.^12^

#### 2.6.2. System suitability

The suitability and reproducibility of the HPLC method were evaluated by analyzing a predetermined BLN concentration (0.125 µg mL^−1^) in six replicates. The suitability of the HPLC method was determined by the retention time and the area under the curve (AUC), which should not exceed 2.0%.

#### 2.6.3. Linearity, range, LOD, and LOQ

Linearity was evaluated by analyzing eight calibration standards ranging from 0.0078 to 1 µg mL^−1^, prepared in triplicate. A linearity graph was plotted of average AUC *versus* concentration (µg mL^−1^). The *R*^2^ value was used to assess linearity, and it should be close to 1. All working standard solutions were stored under refrigeration, away from light. The detection limit (LOD) is the lowest amount that can be detected but not quantified precisely under these conditions, while the limit of quantification (LOQ) is the lowest amount that can be accurately measured.^13^ A statistical approach using a calibration curve close to the limits was employed to determine the detection and quantification limits. LOD and LOQ were calculated using the following equations:
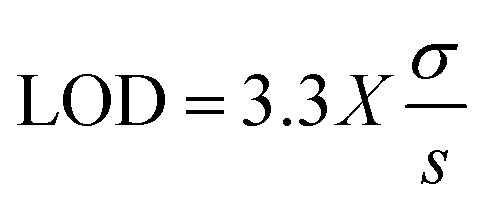

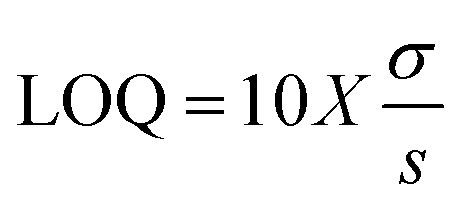
where “*σ*” is the standard deviation of the response and “*s*” is the slope of the calibration curve.

#### 2.6.4. Accuracy

The accuracy of an analytical procedure expresses the closeness of agreement between the accepted value, whether a conventional true value or an accepted reference value, and the value found using the spiking method. 0.125 µg mL^−1^ of BLN working standard solution was spiked by 80%, 100%, and 120%. All the samples were prepared in triplicate and analyzed for drug recovery. The recovery was calculated using the given equation. The discrete and average percent recovery should be within 98% to 102%, and the relative standard deviation (% RSD) should be ≤ 2.0% at each level.
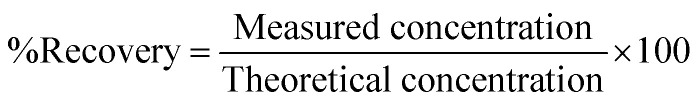


#### 2.6.5. Precision

The precision, defined as the degree of closeness of agreement (scatter) of measurements obtained from multiple samplings of the same homogeneous sample under the prescribed conditions, was assessed as follows: intra-day and inter-day precision were determined as the % RSD of triplicate measurements of BLN quality control (QC) standards. The % RSD was calculated using the equation given below.^14^
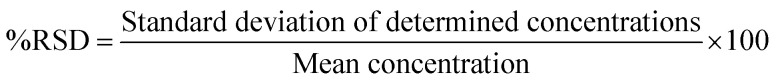


#### 2.6.6. Solution stability

The stability of BLN in the mobile phase was also tested by intermittent injections of the working standard solutions stored at ambient temperature for up to 4 days. The % drug recovery of BLN in the samples before and after storage was compared to determine its stability under the given conditions.

#### 2.6.7. Robustness

The robustness of an analytical procedure is a measure of its capacity to remain unaffected by small, but deliberate variations in method parameters and provides an indication of its reliability during normal usage. The robustness of the developed method was evaluated by purposefully varying chromatographic parameters, such as composition of the mobile phase (±2%), mobile phase pH (3.0 ± 0.2), and flow rate (1.0 ± 0.2 mL min^−1^), among others, for BLN. The influence of these variations on drug quantification, theoretical plate count, and tailing factor was evaluated.

#### 2.6.8. Stress testing or forced degradation studies

The stress testing analysis was performed to evaluate BLN's susceptibility to degradation and to confirm the specificity and stability-indicating potential of the developed analytical method. BLN was stressed under acidic, basic, oxidative, and heat conditions by treating the standard drug sample with 1 mol L^−^^1^ HCl, 1 mol L^−^^1^ NaOH, 30% H_2_O_2_ at 37 °C for 24 hours, and heating at 100 °C for 3 hours, respectively. A neutralizing agent was then added, and a 2-hour waiting period was introduced to ensure complete neutralization before sample processing to avoid any damage to the analytical column (Table 2).^3,15^

**Table 2:** Chromatographic conditions for assay of BLN

Type of degradation	Agent used/condition	Neutralizing agent/2 h
Acidic hydrolysis	1 mol L^−^^^1^^ hydrochloric acid (HCl)/37 °C/24 h	1 mol L^−1^ NaOH
Basic hydrolysis	1 mol L^−1^ sodium hydroxide (NaOH)/37 °C/24 h	1mol L^−1^ HCl
Oxidation	30% Hydrogen peroxide (H_2_O_2_)/37 °C/24 h	30% Sodium Metabisulfite (Na_2_S_2_O_5_)
Thermal stress	100 °C/3 h	

### 2.7. Pharmaceutical applications of the developed analytical method

#### 2.7.1. Solubility studies

The equilibrium solubility of BLN was determined by the shake-flask method, and the results were analyzed using the developed RP-HPLC method. An excess amount of the drug was added to a fixed volume of solvent (water, ethanol, methanol, or isopropyl alcohol) in sealed glass vials. The sealed vials were shaken in a thermostatically controlled water bath at 37 ± 0.5 °C for 24 hours to reach saturation equilibrium. After incubation, the samples were allowed to settle and were centrifuged at 10 000×*g* for 10 minutes. The supernatant was carefully removed, filtered through a 0.22 µm membrane filter, and the first filtrate was discarded to prevent adsorption. The filtrate was diluted with the mobile phase and analyzed by validated RP-HPLC. The drug concentration was calculated from the calibration curve, and solubility was expressed as the mean ± standard deviation (*n* = 3). All measures were taken to avoid exposure of the samples to light and to maintain a constant temperature throughout the experiment.^16^

#### 2.7.2. Formulation preparation and characterization

The BLN-loaded nanoliposomes were made using the solvent evaporation method, with only slight variations from the standard procedure. A solution of 2 mg mL^−1^ BLN in 20 mg mL^−1^ hydrogenated soybean phosphatidylcholine (HSPC) and 5 mg mL^−1^ cholesterol was prepared by dissolving the mixture in methanol and bath sonication. This solution was then added dropwise to 10 mL of preheated water at 65 °C, where the solution was stirred at 1000 rpm for 30 minutes. To separate the macroscopic particles, the solution was first centrifuged at 3000 rpm, and the supernatant was then centrifuged at 15 000 rpm to pellet the nanoparticles. The pellet was then harvested, resuspended in 1 mL of Milli-Q water, and sonicated for 10 minutes. Samples were diluted with filtered Milli-Q water and equilibrated at 25 ± 0.1 °C prior to analysis. Vesicle size and PDI were measured at a backscattering angle of 173° using a 633 nm laser, and the hydrodynamic diameter was calculated based on the Stokes–Einstein equation. Surface charge was measured using a folded capillary cell and calculated from electrophoretic mobility using the Smoluchowski approximation of Henry's equation. A PDI below 0.3 was considered indicative of a uniform dispersion, while a zeta potential magnitude ≥ ±30 mV indicated good colloidal stability. Measurements were performed in triplicate.^17,18^

#### 2.7.3. Entrapment efficiency and drug loading

The HPLC technique developed was used to quantify the amount of BLN entrapped within the final formulation. To release the entrapped drug, the nanoliposomes were disrupted with methanol, releasing BLN into solution, which was subsequently quantified. The entrapment and loading efficiency were calculated using the formula:^19^
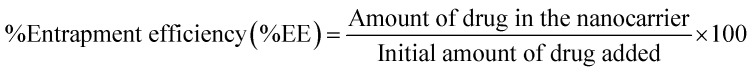

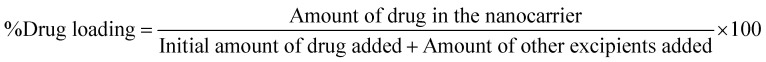


#### 2.7.4. Formulation stability

The stability of the nanoliposomal formulation was evaluated for 1 month by measuring drug retention using the validated RP-HPLC method. Freshly prepared nanoliposomes were lyophilized and stored in airtight amber glass vials under the given storage conditions. At predetermined time points (0, 7, 15, and 30 days), samples were removed and reconstituted with a known amount of distilled water. An aliquot of the sample was disrupted with an appropriate organic solvent to completely release the entrapped drug, followed by vortexing and brief sonication. The solution was then centrifuged at 10 000 rpm for 10 minutes to remove the lipid debris, and the supernatant was filtered through a 0.22 µm membrane filter. The filtrate was appropriately diluted with the mobile phase and analyzed using the validated RP-HPLC method. The drug loading efficiency at each time point was determined from the measured drug amount, and stability was expressed as the percentage of drug retained (mean ± SD, *n* = 3).

### 2.8. Statistical analysis

All experiments were performed in triplicate unless otherwise stated, and results are expressed as mean ± standard deviation (SD). Statistical analysis was performed using OriginPro 2026. Linearity was evaluated using least squares regression analysis. Precision was expressed as a percentage relative standard deviation (%RSD). For solubility studies, statistical significance between groups was assessed using one-way analysis of variance (ANOVA), with *p* < 0.05 considered statistically significant.

## 3. Results and discussions

### 3.1. Preliminary investigation: UV spectroscopic determination

**Fig. 2:**
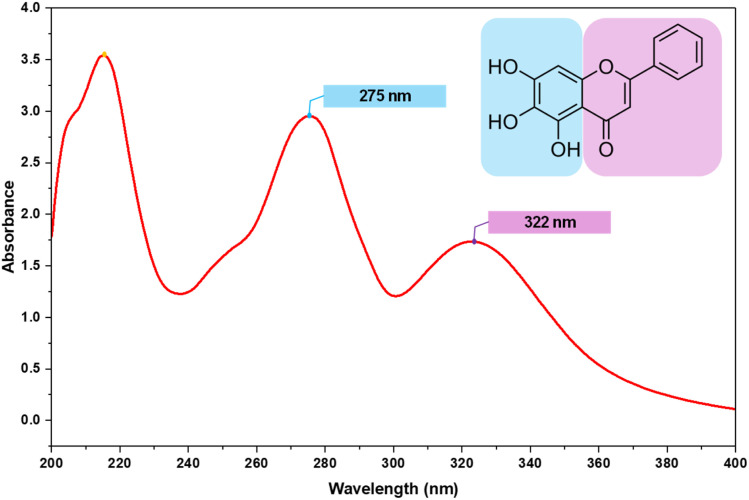
UV spectrum of the standard BLN sample.

In developing an accurate analytical technique, it is crucial to accurately locate the *λ*_max_ of the target compound. As mentioned above, a scan was performed at 10 µg mL^−1^ for each drug over the 200–400 nm range. The *λ*_max_ for BLN was located at 275 nm, as shown in Fig. 2. This value was used for quantification in the development of the RP-HPLC method.

### 3.2. Method development: optimization and validation

#### 3.2.1. Optimization of method parameters

The optimized chromatographic conditions yielded a sharp and symmetrical peak for BLN with a retention time of ∼4.6 min, indicating efficient separation within a short analysis time. Compared with previously reported methods employing phosphate buffer systems or gradient elution, which often result in longer run times and increased solvent consumption, the present method achieves comparable or superior resolution under isocratic, buffer-free conditions. This not only simplifies method operation but also reduces column fouling and environmental burden, aligning with contemporary trends in sustainable analytical method development.

#### 3.2.2. Method validation parameters

##### 3.2.2.1. Specificity

**Fig. 3:**
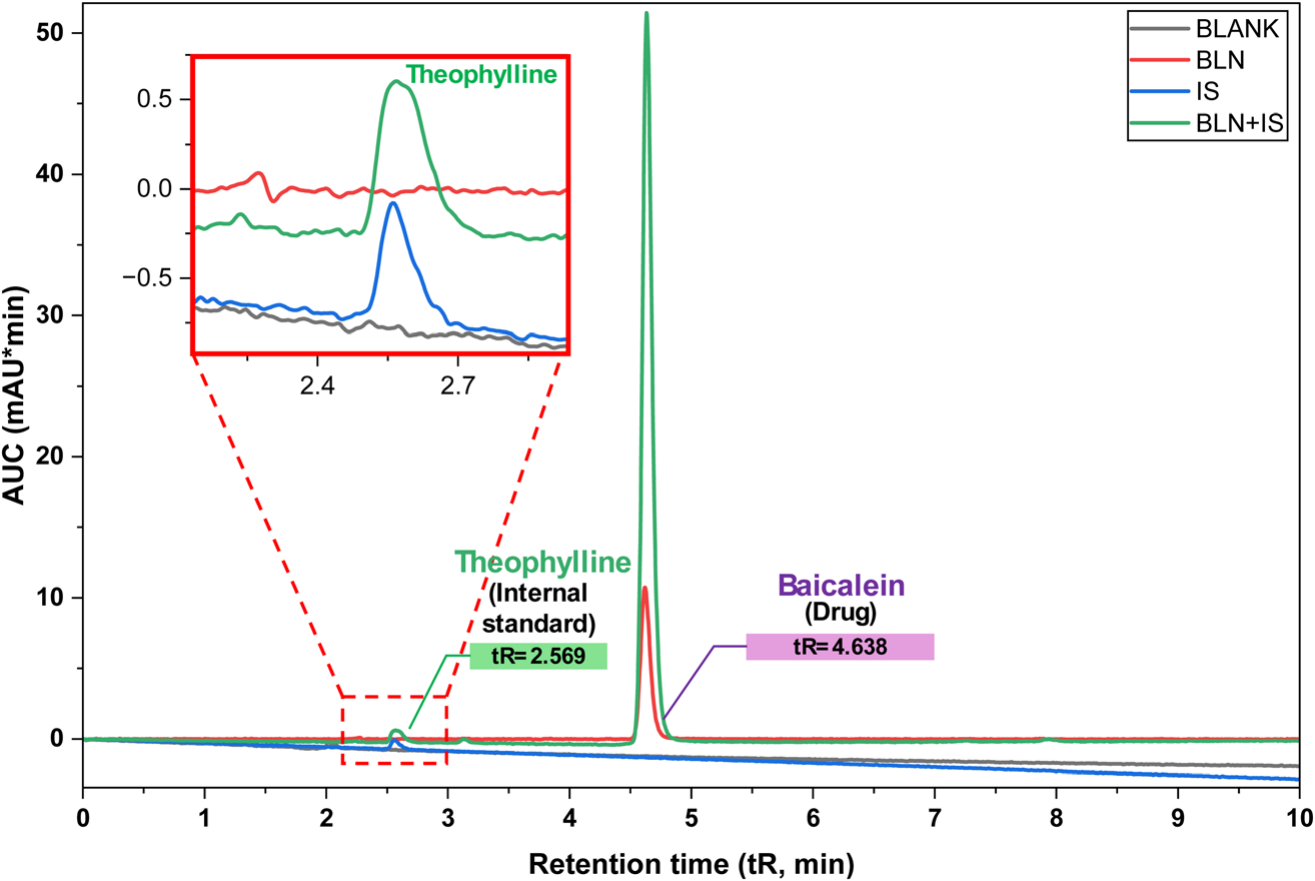
Chromatograms representing specificity in the detection of BLN in the presence of other analytes. The inset shows the method's specificity in accurately differentiating theophylline from BLN in the mixture.

The specificity of the method was demonstrated by injecting the blank (placebo), theophylline (internal standard), baicalein (drug), and a mixture of both. The absence of co-eluting peaks at the BLN retention time confirms the high specificity of the developed method. This underscores the importance of peak purity and spectral confirmation for selectivity in complex matrices. The results demonstrate that the method can accurately distinguish BLN from excipients, internal standards, and degradation products (Fig. 3).

##### 3.2.2.2. Evaluation of system suitability

The HPLC system was subjected to six equal injections of the system suitability solution to evaluate the resolution, retention time, column efficiency, and repeatability. Fig. 3 elucidates the standard chromatogram. The statistical test revealed no significant difference in area or retention time. The % RSD of these parameters was ≤ 2.0%, as presented in Table 3.

**Table 3:** System suitability of the developed method for BLN at 0.125 µg mL^−1^

Sample name	Area (mAU^a^ min)	Retention time (*t*R, min)
BLN-SST-1	0.1307	4.633
BLN-SST-2	0.126	4.637
BLN-SST-3	0.1265	4.633
BLN-SST-4	0.1273	4.637
BLN-SST-5	0.1247	4.637
BLN-SST-6	0.1296	4.637
Mean	0.127466667	4.636
SD	0.002270389	0.002065591
% RSD	1.781162997	0.044558664

a Values represent mean ± SD (*n* = 6), as per ICH Q2 (R2) guidelines.

##### 3.2.2.3. Linearity, range, LOD, and LOQ

**Fig. 4:**
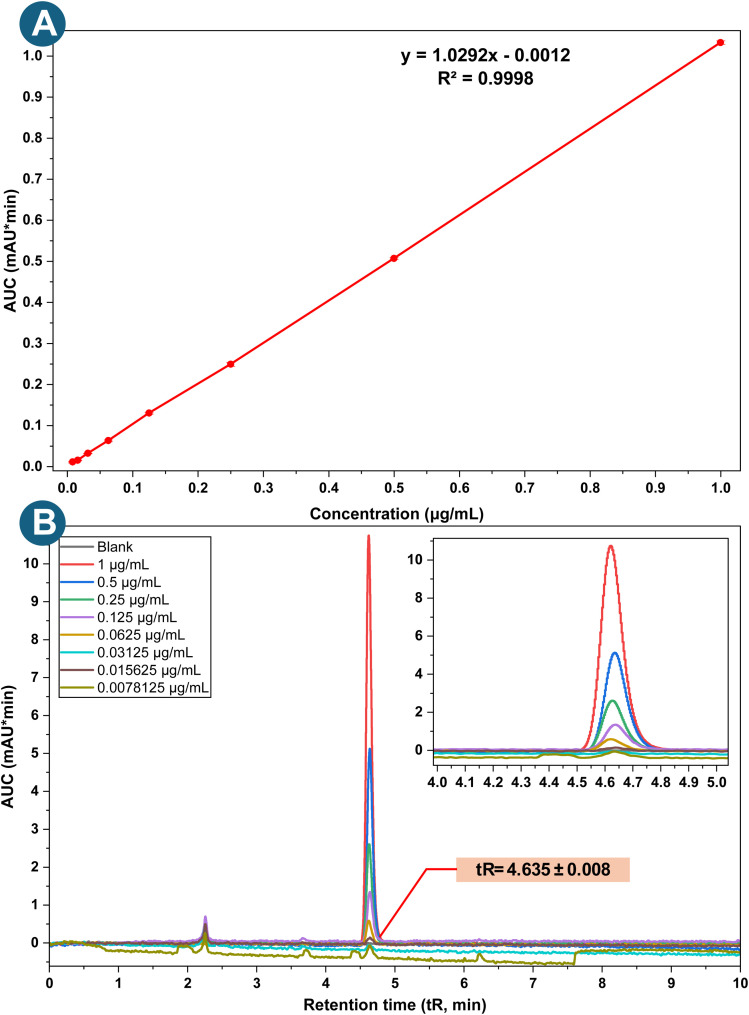
(A) Linearity curve of the calibration standard samples of BLN and (B) graphical representation of the overlay of the calibration standard samples for linearity.

The developed method for quantifying BLN showed linearity over the concentration range of 0.0078–1 µg mL^−1^. The QC concentrations were selected to span the full required analytical range for different quantitation experiments, including drug release, drug entrapment, and permeability. The low QC concentration will provide sensitivity for initial burst-release samples, while the high QC concentration accounts for the maximum amount of unentrapped drug. This ensures that all unknown samples fall within the validated range, enabling precise quantitation. Linear regression analysis yielded an *R*^2^ value of 0.9998 for BLN, indicating extremely high linearity. The linear regression equation for BLN is *y* = 1.0292*x* − 0.0012. The graphical representation of the linear regression equation is presented in Fig. 4. The LOD and LOQ for BLN were 0.0069 and 0.0208 µg mL^−1^, respectively.

The obtained linearity and low LOD/LOQ values demonstrate superior sensitivity compared to the reported HPLC methods for baicalein to date, which have reported higher detection limits due to matrix interference or less-optimized chromatographic conditions. The enhanced sensitivity of the present method is attributed to optimized mobile-phase composition and detection conditions, enabling reliable quantification at trace levels.

##### 3.2.2.4. Accuracy

**Fig. 5:**
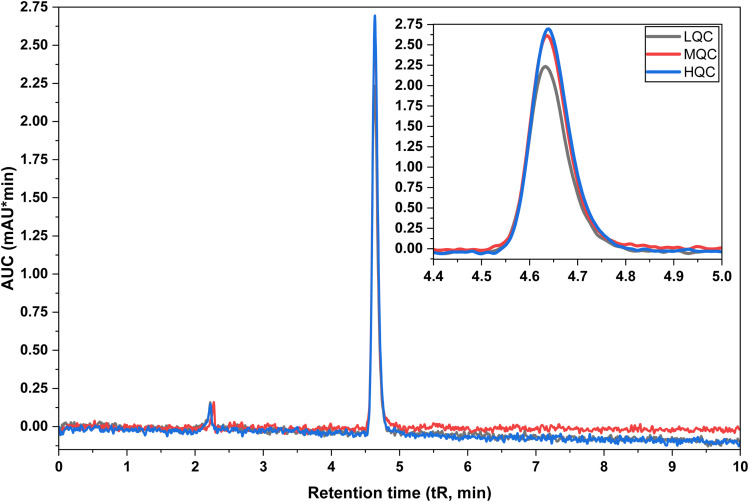
Overlay of chromatograms for LQC, MQC, and HQC samples of BLN.

The accuracy of the developed analytical method was evaluated by spiking BLN with 80%, 100%, and 120% levels of 0.125 µg mL^−1^. Each experiment was performed in triplicate and analyzed for drug recovery (see Fig. 5). The results showed complete recovery of all samples, with an average recovery of 100.00 ± 2.00% and % RSD ≤ 2.00%. The overall mean recovery was 99.26% with a % RSD of 1.32%, indicating high accuracy of the analytical procedure used for quantifying BLN. The results are shown in Table 4. The low recovery variability further confirms the absence of matrix interference and underscores the method's reliability for quantitative analysis in both bulk and formulated systems.

**Table 4:** Accuracy of the developed method^a^

Day 1	% Spike	Conc. (µg mL^−1^)	Conc. (µg mL^−1^)	% Recovery	% RSD
Morning	80	0.225	0.223 ± 0.001	99.063 ± 0.561	0.567
	100	0.25	0.247 ± 0.003	98.769 ± 1.319	1.336
	120	0.275	0.275 ± 0.002	100.048 ± 0.728	0.727
Noon	80	0.225	0.222 ± 0.004	98.631 ± 1.796	1.821
	100	0.25	0.251 ± 0.001	100.285 ± 0.518	0.516
	120	0.275	0.275 ± 0.005	99.754 ± 1.658	1.662
Evening	80	0.225	0.222 ± 0.003	98.833 ± 1.517	1.535
	100	0.25	0.247 ± 0.004	98.653 ± 1.496	1.517
	120	0.275	0.272 ± 0.005	98.977 ± 1.717	1.734

a Values represent mean ± SD (*n* = 3).

##### 3.2.2.5. Precision

The system precision results demonstrated that the % RSD of the BLN peak area was ≤ 2.00%, well within the acceptance criteria. Table 5 provides the intra- and inter-day precision data for BLN, expressed as % RSD. These findings agree with established analytical standards and demonstrate that the method is robust enough for routine pharmaceutical analysis.

**Table 5:** Precision of the developed method^a^

Conc. (µg mL^−1^)	Intra-day precision						Inter-day precision			
	Morning		Afternoon		Evening		Day 2		Day 3	
	% Rec	% RSD	% Rec	% RSD	% Rec	% RSD	% Rec	% RSD	% Rec	% RSD
0.0625	100.68 ± 1.18	1.89	100.64 ± 1.21	1.65	100.57 ± 1.14	1.78	100.27 ± 1.34	1.58	98.77 ± 1.43	1.45
0.25	100.51 ± 0.74	0.737	99.01 ± 0.98	0.99	101.10 ± 1.91	1.94	100.71 ± 1.08	1.27	99.89 ± 0.64	0.65
1	101.73 ± 0.80	0.788	102.68 ± 0.37	0.36	101.45 ± 0.52	0.52	102.17 ± 0.19	0.187	101.99 ± 0.29	0.28

a Values represent mean ± SD (*n* = 9), % Rec. is % recovery.

##### 3.2.2.6. Solution stability

The stability of the stock solution at 1 µg mL^−1^ was determined over 4 days by comparing the % drug recovery of the QC samples before and after storage at room temperature (Table 6). No significant changes in BLN recovery were observed, and no degradation peaks were observed. This result indicates that BLN is stable in the mobile phase for 4 days at room temperature.

**Table 6:** Solution stability of the developed method^a^

Time (h)	% Recovery/% RSD
24	100.437 ± 0.399/0.397
48	101.937 ± 0.471/0.462
72	102.170 ± 0.191/0.187
96	101.992 ± 0.293/0.287

a Values represent mean ± SD (*n* = 3).

##### 3.2.2.7. Robustness

**Fig. 6:**
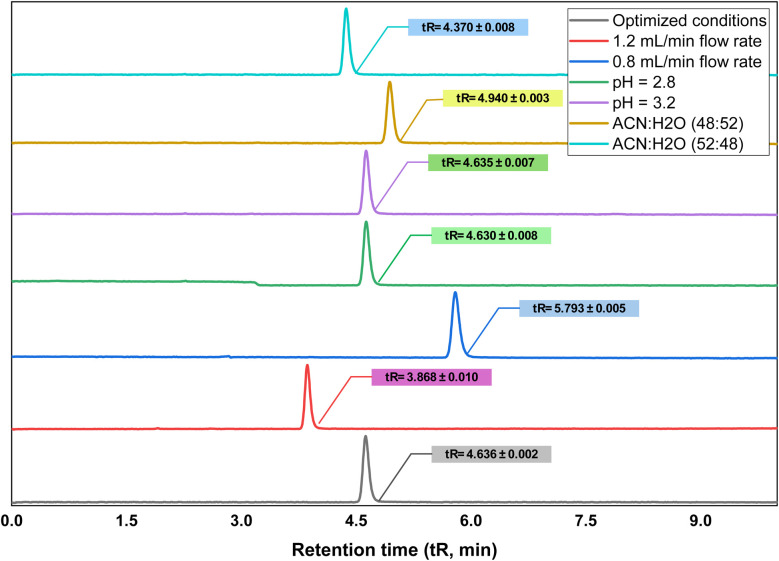
Chromatograms representing the change in the tR with changes in the optimized conditions.

The robustness of the proposed method was assessed by comparing the results with those of the standard BLN solution, and only slight differences were observed (Table 7 and Fig. 6). Even with intentional parameter changes, the optimized method demonstrated insignificant differences in the retention time, tailing factor, and theoretical plates. The system suitability factors were met under all robustness conditions, and the BLN peak tR remained unchanged. The BLN assay results remained within the acceptance limits. The differences in flow rate (±0.2 mL min^−1^), mobile phase composition (±2%), and pH (±0.2 pH units) were within the acceptance criteria, demonstrating the method's robustness. The method is robust.

**Table 7:** Robustness of the developed chromatographic method^a^

Flow rate	pH of the mobile phase		Mobile phase composition (ACN : water)	
	3.0		50 : 50	
Parameters	Retention time	Peak area	Tailing factor	Theoretical plates
**Optimized condition**				
1.0 mL min^−1^	4.636 ± 0.002	1.033 ± 0.004	1.27 ± 0.016	15 718 ± 91

**Change in flow rate**				
0.8 mL min^−1^	5.793 ± 0.005	1.277 ± 0.014	1.3 ± 0.006	16 463 ± 73
1.2 mL min^−1^	3.868 ± 0.010	0.877 ± 0.005	1.2 ± 0.021	14 758 ± 87

**Change in the pH of the mobile phase**				
2.8	4.630 ± 0.008	1.032 ± 0.046	1.29 ± 0.020	15 660 ± 113
3.2	4.635 ± 0.007	1.005 ± 0.009	1.24 ± 0.017	15 801 ± 33

**Change in mobile phase composition ACN : water**				
48 : 52	4.940 ± 0.003	1.002 ± 0.004	1.21 ± 0.021	16 358 ± 210
52 : 48	4.370 ± 0.008	1.008 ± 0.005	1.29 ± 0.011	15 288 ± 45

a Values represent mean ± SD (*n* = 3).

##### 3.2.2.8. Forced degradation studies

**Fig. 7:**
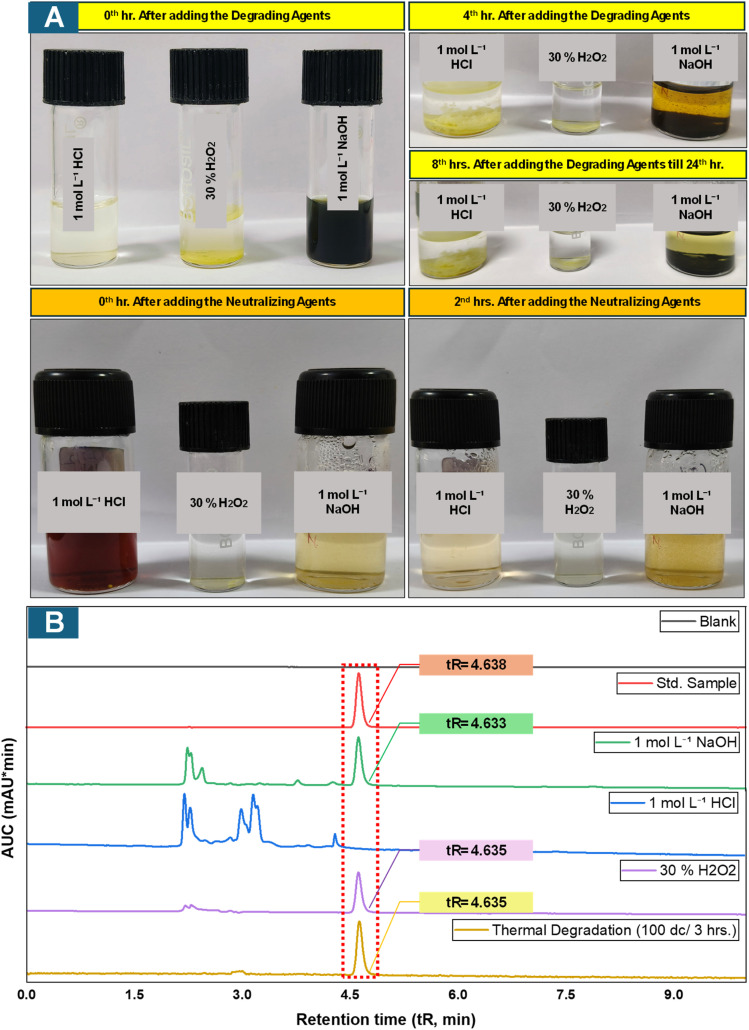
(A) Photographs of the incubation of the standard drug sample in various degradation media and their respective physical changes over the whole 24 h before neutralization for 2 hours. (B) Overlay of chromatograms for the forced degradation samples of BLN.

Forced degradation studies were performed to assess the inherent stability of BLN and to understand its degradation characteristics under various chemical and thermal stress conditions. To the best of current knowledge, no previous study has systematically reported the degradation characteristics of BLN under various stress conditions. Although a few previous studies have indicated degradation under alkaline conditions, these views are not supported by any analytical data. The findings of the current study contradict these views. BLN samples were stressed under acidic (1 mol L^−1^ HCl), basic (1 mol L^−1^ NaOH), and oxidative (30% H_2_O_2_) conditions for 24 h at 37 °C, and under thermal stress at 100 °C for 3 h. Under acidic conditions, complete hydrolysis of BLN was observed, which was evident by the disappearance of the parent chromatographic peak and the absence of the retention time corresponding to the standard. However, partial hydrolysis was observed under alkaline conditions, with the predominant peak resembling the standard, indicating greater stability than previously thought. Moreover, negligible degradation was observed under oxidative and thermal stress conditions, indicating high resistance against peroxide-induced oxidation and elevated temperature (Fig. 7(A) and (B)). Taken together, these observations suggest a high susceptibility of BLN to acidic degradation, suggesting potential instability at gastric pH and, therefore, a limitation for direct oral delivery. However, low degradation even under strong alkaline conditions suggests adequate stability at mildly basic physiological environments. The forced degradation study provides valuable information for formulation development, storage, and handling practices, and also confirms the stability-indicating specificity of the developed chromatographic method for its intended use in analytical and bioanalytical studies. Compared with earlier studies without degradation profiling, the present work provides a more comprehensive evaluation, confirming the method's stability-indicating capability and its applicability in formulation development and stability studies.

### 3.3. Novelty of the method

The comparative analysis indicates that previously reported HPLC methods for baicalein predominantly employ buffer-based mobile phases, gradient elution, and multi-solvent systems, often resulting in longer retention times (6.6–60.41 min) and increased analytical complexity. While certain methods offer enhanced sensitivity, particularly µHPLC-ECD they require specialized instrumentation or extensive solvent use. In contrast, the present method utilizes a buffer-free, isocratic system with a significantly reduced retention time (∼4.6 min), while maintaining adequate sensitivity (LOD 0.0069 µg mL^−1^) and excellent linearity. Furthermore, unlike most reported methods focused on phytochemical or pharmacokinetic analysis, the proposed method uniquely integrates stability-indicating capability and applicability to nanopharmaceutical systems, thereby offering a more sustainable, efficient, and application-oriented analytical platform. A comparative analysis of the reported methods has been summarized in Table 8.

**Table 8:** Comparative analysis of the HPLC methods reported for BLN^a^

Analytes	Mobile phase	Mode	tR_BLN_ (min)	Linearity range (µg mL^−1^)	LOD/LOQ (µg mL^−1^)	Detector	Key features	Limitations *vs.* present method	Ref.
*Baicalein*	*Water (pH 3, OPA): ACN (50 : 50)*	*Isocratic*	*∼4.6*	*0.0078* *–1 *	*0.0069/0.0208*	*HPLC-* *DAD*	*Green, stability-indicating, nano-applicable*	*Moderate sensitivity compared to ECD-based methods*	*Present method*
Baicalein, paclitaxel	MeOH : Milli Q water (with 0.1% FA) (30 : 70)	Isocratic	4.3	0.019–10	0.119/0.361	HPLC-UV	Simultaneous dual drug estimation	Isocratic elution and higher organic solvent consumption	6
Baicalein, chrysin, wogonin	MeOH-ACN-Milli Q water: OPA (30 : 38 : 60 : 1)	Isocratic	6.6	0.5–400	0.4/1.2	HPLC-UV	Multi-flavonoid analysis	Complex mobile phase, longer runtime	7
Baicalein, baicalin	ACN-Milli Q water (40 : 60) (with 0.05% OPA)	Isocratic	20	0.08–10	0.08/0.04	HPLC-UV	Pharmacokinetic analysis	Higher organic solvent consumption, matrix complexity	8
Baicalein, baicalein-7-glucuronide	MeOH-ACN-phosphate buffer	Gradient	15	1–10	0.03/0.05	HPLC-UV	Metabolite profiling	Gradient system, longer analysis time, buffer use	9
Baicalein, baicalin	MeOH- Milli Q water (with 0.5% OPA)	Isocratic	9.1	1.4 pg to 2.7 ng	0.54 pg/14 pg	Micro HPLC (µHPLC)-ECD	High sensitivity detection	Requires specialized detector	10
Baicalein	ACN/Milli Q water/FA (21/78/1, v/v/v): ACN/Milli Q water/FA (80/19/1, v/v/v)	Gradient	60.41	NR	3.75 ng/12.5 ng	HPLC-DAD	Extremely high sensitivity	Extremely long runtime, high solvent consumption	20
Baicalein, phytochemicals	MeOH-ACN-Milli Q water (with 1% OPA)	Isocratic	12.31	5.3–37.1	0.546/NR	HPLC-UV	Herbal matrix analysis	Longer runtime	21

a Where, NR – not reported, FA – formic acid, MeOH – methanol, pg – picograms, ng – nanograms.

### 3.4. Application of the analytical method in pharmaceutical formulation

#### 3.4.1. Solubility studies

**Fig. 8:**
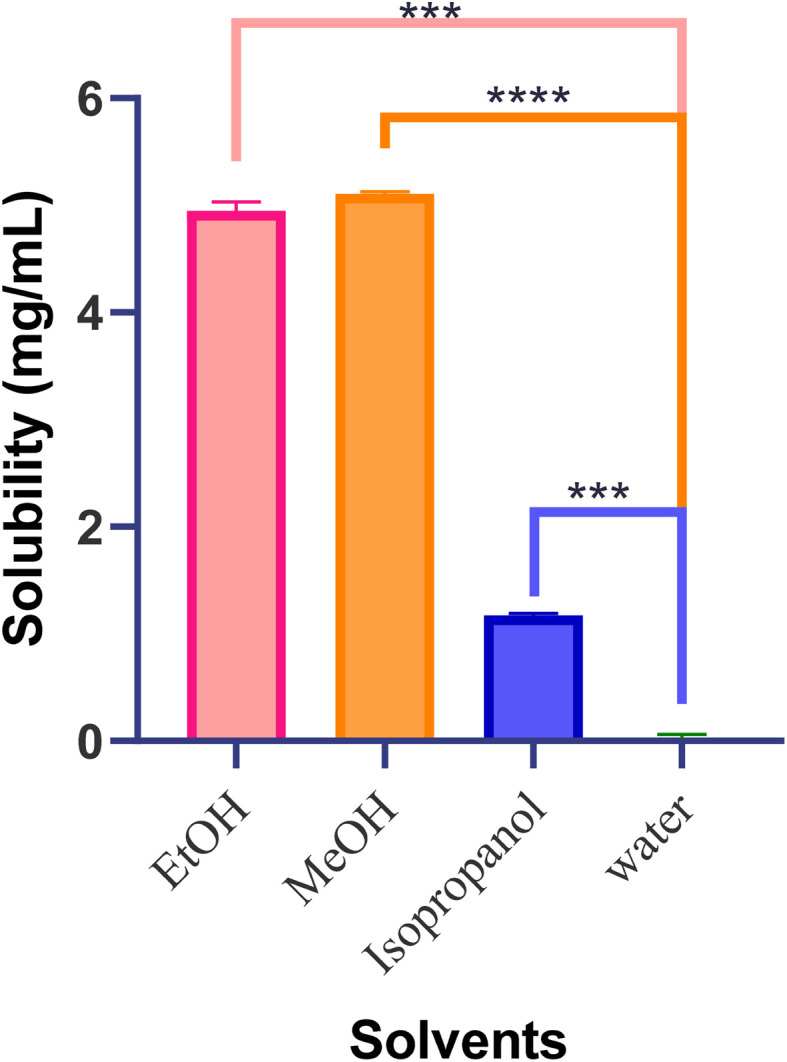
Graphical representation of the solubility of BLN in water as compared to various organic solvents. Values represent mean ± SD (*n* = 3).

The solubility profile of BLN in water and other organic solvents was quantitatively evaluated using the developed reverse-phase high-performance liquid chromatography (RP-HPLC) method. The data clearly show that BLN has extremely poor solubility in water (<0.1 µg mL^−1^) and higher solubility in organic solvents, which correlates with its high lipophilicity (Fig. 8). The use of a sensitive and specific HPLC assay enabled precise measurement of low BLN concentrations in aqueous solutions, a task difficult to achieve with standard spectrophotometric analysis due to interference and limited sensitivity.

The results of the repeated-measures ANOVA showed that solvent selection significantly influenced the analytical response (*p* < 0.0001), with exceptionally low replicate variance, indicating high experimental reproducibility. The post-hoc analysis revealed that all the organic solvents produced significantly higher responses than water, with the order of magnitude being methanol ≈ ethanol ≫ isopropanol ≫ water.

The solubility profiling has important pharmaceutical implications, establishing that BLN belongs to the poorly water-soluble class of compounds, underscoring the need for solubility enhancement approaches for successful formulation. The solubility data directly support the choice of lipid-based and vesicular carrier systems, such as liposomes, where drug distribution into the lipid bilayer can enhance solubility, stability, and bioavailability. Thus, the developed HPLC method serves not only as a quantitative analytical technique but also as a valuable preformulation tool necessary for rational formulation development.

The observed poor aqueous solubility of BLN is consistent with its classification as a BCS Class II compound and aligns with previously reported literature. The significantly higher solubility in organic solvents further confirms its lipophilic nature. Similar findings have been reported in studies that employed lipid-based and vesicular systems to enhance the solubility and bioavailability of poorly water-soluble drugs. These results strongly justify the selection of nanoliposomal carriers for improving BLN delivery.

#### 3.4.2. Formulation preparation and characterization

**Fig. 9:**
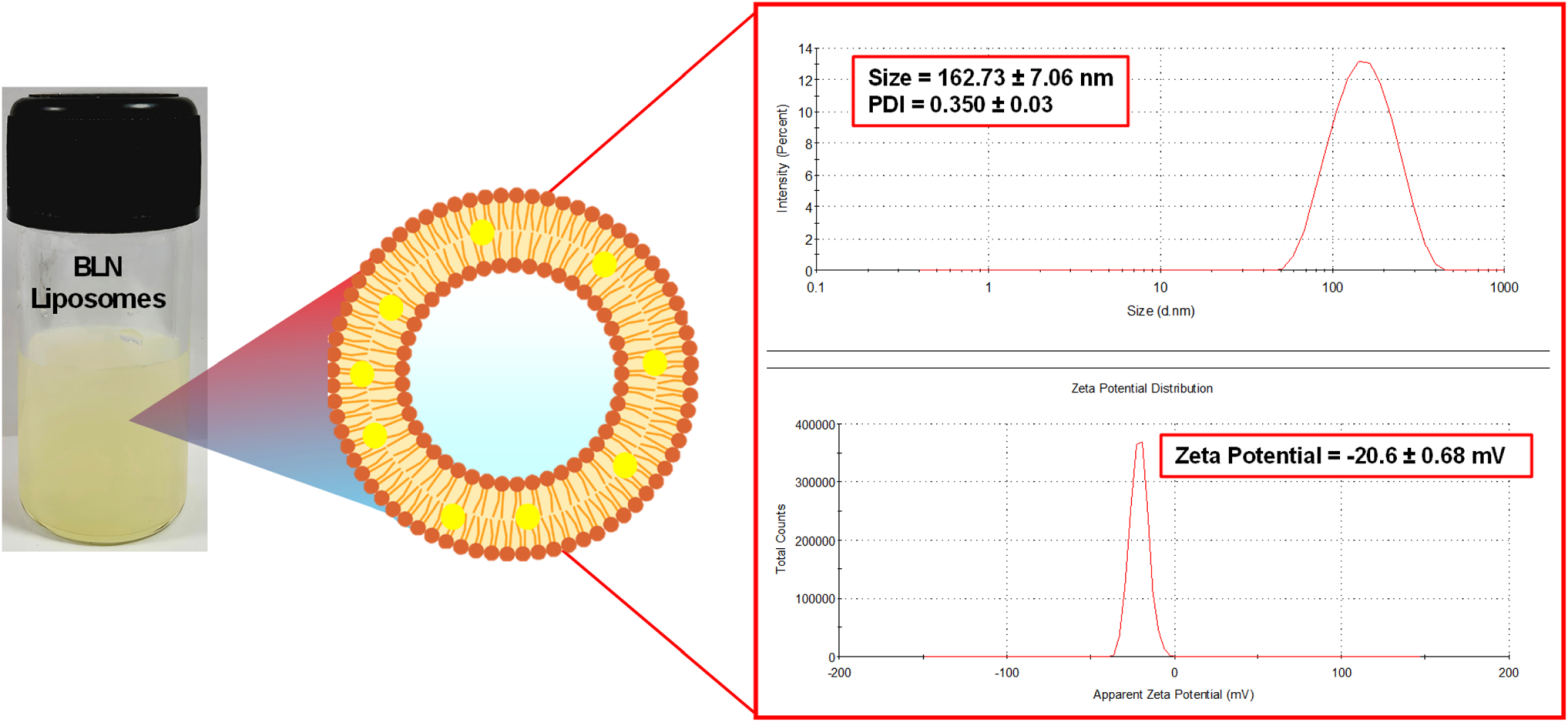
Photographic representation of the prepared nanoliposomal formulation, along with size and zeta potential characterization. Values represent mean ± SD (*n* = 3).

BLN-loaded nanoliposomes were successfully prepared using a modified solvent evaporation technique. The resuspended nanoliposomes were characterized for their size, PDI, and zeta potential, which were found to be 162.73 ± 7.06 nm, 0.350 ± 0.03, and −20.6 ± 0.68 mV, respectively. The values indicate a relatively uniform nanodispersion within the acceptable range reported in the literature for liposomal drug delivery systems. Although the zeta potential (∼−20 mV) suggests moderate electrostatic stability, similar values have been observed in phospholipid-based systems where steric stabilization contributes significantly to overall stability. These characteristics confirm the formulation's suitability for drug delivery applications (Fig. 9).

#### 3.4.3. Drug entrapment and drug loading of the nano-liposomes

The developed technique was used to determine the entrapment and loading efficiencies of the nanoformulation. The entrapment efficiency of BLN was 62.2 ± 0.040%. The loading efficiency of the formulation was calculated to be 5.92 ± 0.016% BLN. In the absence of any observable effect from the formulation components, the developed technique demonstrates accuracy in measuring the BLN in the given nanoformulation. This level of encapsulation can be attributed to favourable drug–lipid interactions and partitioning of BLN within the lipid bilayer. The results demonstrate that the developed formulation is efficient in incorporating poorly soluble compounds.

#### 3.4.4. Stability of the developed nanoliposomal formulation

The lyophilized nanoliposomal formulation exhibited no significant change in drug content over the one-month study period. The percentage of drug retained remained within acceptable limits, indicating that the formulation maintained its integrity and was stable for at least 30 days under the studied storage conditions (Table 9). These findings further validate the applicability of the developed RP-HPLC method for monitoring formulation stability.

**Table 9:** Stability studies of the developed nanoliposomal formulation of BLN^a^

Time (Days)	Drug loading (%)
0	5.92 ± 0.016
7	5.65 ± 0.003
15	5.32 ± 0.021
30	5.54 ± 0.046

a Values represent mean ± SD (*n* = 3).

Overall, the developed RP-HPLC method demonstrated excellent analytical performance, including high sensitivity, precision, accuracy, and stability-indicating capability. A comparative evaluation with the existing literature highlights its advantages, including reduced analysis time, elimination of buffer systems, and improved environmental compatibility. Furthermore, the successful application of the method in nanoliposomal systems underscores its versatility in both analytical and formulation domains. The integration of multi-metric greenness assessment further strengthens its relevance in modern sustainable analytical chemistry. Collectively, these findings establish the method as a robust, eco-conscious, and application-oriented analytical tool with significant potential for routine pharmaceutical analysis and advanced drug delivery research.

## 4. Greenness assessment for the developed analytical method

The environmental sustainability of the analytical procedure before its application in the laboratory is an important consideration aimed at reducing the discharge of hazardous materials. Currently, there has been a strong focus on prioritizing the greenness of analytical procedures, and reliable methods are now available to assess the environmental sustainability. The current study uses six methods to validate the environmental sustainability of the developed method: the MoGAPI,^22^ VIGI,^23^ CaFRI,^24^ CACI,^25^ BAGI,^26^ and the AGREE.^27–29^

### 4.1. BAGI assessment

**Fig. 10:**
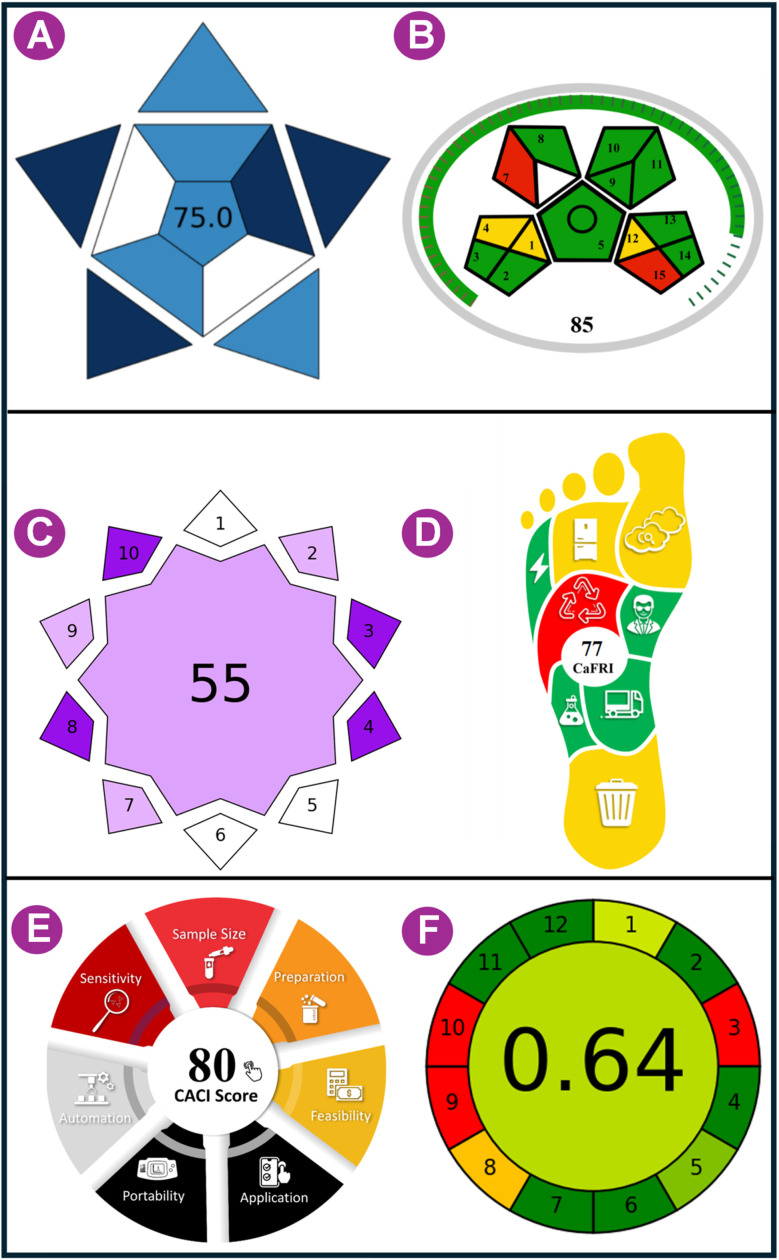
Blueness of the developed RP-HPLC method was evaluated by (A) BAGI and Greenness by (B) MoGAPI, (C) VIGI, (D) CaFRI, (E) CACI, and (F) AGREE scores.

The BAGI tool, used in conjunction with the Perfect Green Indicator, assesses the applicability and functionality of an analytical technique using 10 defined parameters. The pictorial representation in the BAGI tool exemplifies the process, divided into phases: characteristics 1–3 (analytical determination), characteristics 4–5 (sample preparation), and characteristics 6–10 (analytical procedure). A total score of 60 or more is required to define the method's pertinence. The developed method yielded a score of 75 (Fig. 10(a)).

### 4.2. MoGAPI

The Green Analytical Procedure Index (GAPI), launched in 2018, evaluates the sampling and analysis phases of analytical procedures. As GAPI does not have a complete numerical greenness score, it was modified in 2024 to MoGAPI (Modified GAPI). GAPI has a five-point color-coded pictogram for the environmental effect of each step, while MoGAPI gives a numerical pentagram score for easier evaluation. A score of ≥75 is “excellent green”. In the current work, MoGAPI assigned the proposed method a greenness score of 85, indicating a highly environmentally compatible approach (Fig. 10(b)).

### 4.3. VIGI tool

To provide a comprehensive evaluation, in addition to the existing green, blue, and red rankings, VIGI combines ten criteria: sample preparation and instrumentation, data processing and software, white analytical chemistry and derivatives, regulatory compliance, materials and reagents, miniaturization, automation, interdisciplinarity, sensitivity, and approach. The method's innovation score is represented by a star-shaped decagon in a survey. The overall VIGI score, ranging from 1 to 10, is calculated as the geometric mean of all criteria scores. VIGI received a total score of 55, giving emphasis to its distinctiveness, as shown in Fig. 10(c).

### 4.4. CaFRI

The Carbon Footprint Reduction Index (CaFRI) is an online, free tool that evaluates and reduces the carbon footprint of analytical procedures in chemical labs, with carbon footprint as the primary concern and accounting for both method requirements and laboratory-specific information. The result is a value from 0 to 100 (77 at present). The main parameters are energy consumption (total electrical energy and number of samples), energy production emissivity, carbon footprint reduction methods, chemical consumption, sample storage and transportation, employee participation, and waste management/recycling. CaFRI is a questionnaire-based tool that estimates these parameters and displays the results using a human-foot pictogram in red (low), yellow (medium), and green (good) colours to represent performance and encourage emission reduction, connecting analytical chemistry with sustainability (Fig. 10(d)).

### 4.5. CACI

The evaluation is intended to assess the feasibility and usability of established analytical methods. Unlike conventional parameters, the CACI provides a comprehensive analysis of operational factors, including sample size, preparation, feasibility, applicability, portability, sensitivity, and automation. The results are presented using a diagnostic pictogram, where coloured areas indicate excellent performance, grey areas indicate moderate performance, and black areas indicate non-compliance or failure to meet criteria. The resultant score obtained was 80 (Fig. 10(e)).

### 4.6. AGREE

Green designable software was employed to evaluate agreement with the 12 green principles of analytical chemistry. The software program provides a final score ranging from 0 to 1 using a 12-sector pictogram. The colours of the sectors (green, yellow, red) indicate the relative importance and score of each criterion, with darker colours indicating higher importance. For the new RP-HPLC method, the pictogram score was 0.64 (Fig. 10(f)), indicating its eco-friendliness.

Overall, the sustainability of the developed RP-HPLC method was assessed using six different tools, and the results confirmed its superior greenness. The method scored 75 on the BAGI, which is adequate for operational feasibility, and has a streamlined analytical process with less sample preparation. The MoGAPI score of 85 categorized the method as “excellent green” due to the solvent's low toxicity, low consumption, and low hazardous-waste generation. The VIGI score of 55 indicated that the method is innovative and advanced while maintaining regulatory compatibility and automation. The CaFRI value of 77 supported the reduction in carbon emissions due to shorter analysis time, ambient conditions, and lower energy use. Additionally, the high CACI score of 80 supported the method's high usability, sensitivity, and applicability in the laboratory. Finally, the AGREE score of 0.64 supported the method's high compliance with the twelve principles of Green Analytical Chemistry. Taken together, the results show that the developed method combines environmental sustainability with analytical efficiency and feasibility, making it a highly efficient green analytical method for pharmaceutical analysis.

## 5. Conclusions and outlook

An RP-HPLC method has been developed and validated for the quantitative estimation of BLN in accordance with ICH guidelines, covering specificity, linearity, precision, accuracy, LOD/LOQ, robustness, and stability-indicating capability. The forced degradation study under various stress conditions demonstrated the method's ability to selectively separate BLN from its degradation products, thereby establishing its suitability for stability studies. The standard and sample solutions were found to be stable for up to 4 days at room temperature, thus establishing the method's reliability for routine analytical work. The validated method establishes it as a reliable tool for BLN integrity, potency, and consistency testing during its shelf life and for routine use in pharmaceutical quality control laboratories. The method has wider applications in formulation development and preformulation studies. Despite these advantages, certain limitations should be acknowledged. The use of acetonitrile contributes to a moderate environmental footprint. Additionally, the study is limited to laboratory-scale validation and specific nanoformulation systems, and further work is required to assess scalability and broader applicability. The method's stability-indicating capability enables monitoring of BLN degradation across various formulations, including suspensions, nanoparticles, liposomes, and solid dispersions, thereby facilitating optimization of excipient compatibility and storage conditions. The method can also be used in solubility enhancement studies to estimate BLN in various solubilizing agents, such as cosolvents, surfactant systems, complexation systems, and lipid-based carriers, thereby allowing the selection of appropriate drug delivery systems. In the future, the method can be used for dissolution profiling, *in vitro* release kinetics, degradation pathway studies, and scale-up process monitoring, and can be used as a model analytical method for bioanalytical method adaptation or therapeutic product development for BLN. Currently, our lab is also working on the solubility enhancement strategies of BCS class II drugs, including BLN.

## Author contributions

Sweta Acharya: data curation, investigation, writing – original draft, writing – review and editing, methodology, software, validation. Ankit Jain: project administration, supervision, writing – original draft, writing – review and editing.

## Conflicts of interest

There are no conflicts to declare.

## Abbreviations

% RSD: % Relative standard deviation
ACN: Acetonitrile
AGREE: Analytical GREEnness
AUC: Area under curve
BAGI: Blue applicability grade index
BA: Bioavailability
BCS: Biopharmaceutical classification system
API: Active pharmaceutical ingredient
BDS: Base-deactivated silica
BLN: Baicalein
CACI: Click analytical chemistry index
CaFRI: Carbon footprint reduction index
DAD: Diode array detector
H_2_O_2_: Hydrogen peroxide
HCl: Hydrochloric acid
HQC: Higher quality control
HSPC: Hydrogenated soy phosphatidylcholine
ICH: International council for harmonisation of technical requirements for pharmaceuticals for human use
LOD: Limit of detection
LOQ: Limit of quantification
LQC: Lower quality control
MoGAPI: Modified green analytical procedure index
MQC: Middle quality control
Na_2_S_2_O_5_: Sodium metabisulfite
NaOH: Sodium hydroxide
OPA: Orthophosphoric acid
PDI: Polydispersity index
QC: Quality control
RP-HPLC: Reversed-phase high-performance liquid chromatography
*t*R: Retention time
UV: Ultra-violet
VIGI: Violet innovation grade index

## Data Availability

The data supporting the findings are presented within the paper, and additional data may be requested from the authors.
